# Geological exploration of coal mine burnt rock and waterlogged area boundary based on transient electromagnetic and high-density electrical resistivity

**DOI:** 10.1038/s41598-024-55496-6

**Published:** 2024-03-01

**Authors:** Yanlong Yang, Ci Zhao, Yaxiao Di, Qian Li

**Affiliations:** 1 China Building Materials Industrial Geological Exploration Center Xinjiang Corps, Urumqi, 830000 China; 2 Chinese Research Academy of Environmental Sciences, Beijing, 100012 China; 3 Xinjiang University, College of Geography and Remote Sensing Sciences, Urumqi, 830017 China

**Keywords:** Hydrology, Hydrogeology

## Abstract

The water-rich burnt rock may threaten the safe production of coal mines. Identifying the boundaries of burnt rock and the water-rich area is of great practical significance for the ensuring the safety of mining operations. Transient electromagnetic and high-density resistivity methods are commonly employed in geophysical exploration, such as for investigating the presence of groundwater or delineating boundaries of altered rocks. These methods are non-invasive and provide detailed information about subsurface conditions without the need for drilling or excavation. The Jiangjun Gobi No. 1 open-pit coal mine is situated in the Kalamaili fault zone and is characterized by a high groundwater content. In certain mining sites within the study area, the inflow of water reaches tens of thousands of cubic meters per day, which significantly impacts production and presents major risks. To accurately determine the boundaries of burnt rock and water accumulation areas in the Jiangjun Gobi No. 1 open-pit coal mine, this paper uses different geophysical prospecting methods based on the depth of the strata. The middle and deep parts are investigated using the high-resolution transient electromagnetic method, while the shallow parts are examined using the high-accuracy high-density electric method. Through analyzing the electrical characteristics of the study area, it is inferred that the low-resistivity area in the northwest represents a shallow surface water-rich region. This area extends continuously towards the northwest, is not trapped, and is supplied by surface water. The deep low-resistivity zone primarily consists of sandstone and coarse sandstone. It is inferred that the low-resistance area in the southern part of the study area is also a shallow surface water-rich region, extending towards the east and west sides, not trapped, and supplied by surface water. The deep low-resistivity zone mainly comprises a combination of sandstone, coarse sandstone, and burnt rock, with intermittent layers of mudstone and argillaceous sandstone. The boundary line of burnt rock (coal-bearing strata) is located in the south of the study area. The resistivity of burnt rock (coal-bearing strata) is higher than that of the surrounding rocks, and the resistivity of coal seams is slightly higher than that of sandstone with larger porosity. Estimating the boundaries of groundwater and altered rocks serves to prevent geological disasters and provides valuable information for mineral development and ecological protection.

## Introduction

Burnt rocks rich in water may threaten the safe production of coal mines, making it crucial to identify the boundaries of burnt rocks and water-rich areas for safe mining operations. When water-filled fractures occur in the coal-bearing sandstone or when a fractured zone is intersected by a fault containing water, the high electrical conductivity of the water creates a noticeable contrast with the surrounding rock structure. This serves as the geophysical basis for hydrogeological exploration using electromagnetic methods. The high-density electrical method finds wide applications, primarily in karst disaster investigation, reservoir dam foundation leakage exploration, geological exploration in highway viaducts and karst area, pipeline detection, water geophysical prospecting, high-rise building site selection, and coalfield collapse detection. In recent years, its application in stone exploration has also been gradually developed, providing valuable reference materials for mining operations. The high-density resistivity method is a cost-effective, efficient, and information-rich electrical prospecting technique. It offers convenient interpretation and significantly enhances exploration capability. The DC resistance method can detect groundwater during the process of coal mine excavation^1^. Integrated geophysical detection, transient electromagnetic, and high-density electrical resistance methods can detect water accumulation in goafs (gob areas) of coal mines^2^. Xue et al. conducted a literature review on electrical and electromagnetic methods for coal mine detection in China^3^. With the rapid development of China’s economy in the past 20 years, accurate detection of water-rich areas and hidden aqueducts in goafs has become a new goals in recent years. Niu et al. tested and analyzed the characteristic response of electric potential signal in coal seam mining activities^4^. Limestone water eruption may cause major disasters during the development of deep coal mines. Zhu et al. verified and determined the true distribution of grouting parameters based on the finite element method of COMSOL Multiphysics. The results show that the design meets the engineering requirements^5^. The transient electromagnetic method can be used to detect mined-out areas in mining areas filled with accumulated water^6^.

Yu et al. reviewed the geophysical mapping of coal mine water structure and analyzed the current challenges^7^. Multi-angle transient electromagnetic requires a 2.5-D inversion method. Chen et al. used the 2.5-D least squares inversion method to detect water inrush on the side of a coal mine road in Shandong, China. The inversion results were verified by comparing them with the data from boreholes and excavation. The 2.5-D inversion method has better interpretation accuracy and resolution than the 1-D inversion method^8^. Electrical Resistivity Tomography (ERT) can be used to identify waterways in old mining areas after the rainy season in India^9^. Electrical Resistivity Tomography (ERT) can be used to detect the location of sinkholes^10^ and can also be used to detect underground cavities^11^. The method combining self-potential, electrical resistivity tomography and induced polarization is used in the detection of gold mines in India^12^. The particle swarm optimization-GIS inversion algorithm (PSO-GIS) is applied to the measurement of coal mines, and its results are consistent with those obtained through drilling verification. The PSO-GIS algorithm can improve the interpretation effect of the full space inversion of coal mines^13^. Particle swarm optimization algorithm and simulated quenching inversion algorithm were used by Kumar et al. to analyze self-potential (SP) data, and then Electrical Resistivity Tomography (ERT) was used to perform detailed high-resolution mapping of seepage and drainage in coal mines^14^. The self-potential (SP) method is a low-cost, non-invasive passive geophysical technique, and Sahadev et al. used the self-potential (SP) method to detect coal fires below the surface^15^. Li et al. detected the Dongsheng coal mining area in Inner Mongolia based on the combined surface-drilling DC resistivity method, and the changes in the resistivity response characteristics of water diffusion were effectively detected^16^. Luo et al. utilized the Ground Penetrating Radar (GPR) detection method to assess the soil moisture, soil layer thickness and soil gravel content in the mining area, and provide scientific data for the reclamation of the mining area^17^. Saurabh et al. employed Self-Potential (SP) and Electrical Resistivity Tomogrphy (ERT) technologies to determine the position, depth, range, and state of the working channel, and analyzed the SP data using simulated quenching optimization technology. ERT data were acquired using Wenner, Dipole–Dipole and Schlumberger matrices. The inversion is performed using 2.5D ZZRESINV software, and the overall results are consistent with lithological materials and ground photography results^18^. Sun et al. probed the upper limit of an ultra-thick unconsolidated aquifer in Huainan^19^. Two new indicators were constructed to assess coal fire risk, one is Dynamic Load Index (DLI), and the other is dynamic-static loading evaluation index^20^. Abandoned mine tunnels filled with water at unknown locations may pose potential risks to safe production in modern mines, and underground transient electromagnetic methods can detect water-filled tunnels. The 3D finite-difference time-domain modeling method can be used to simulate the anomalous response and parameter influence of water-filled tunnel junctions^21^. Some literatures have reviewed the geophysical detection methods of mined-out areas and rich water in mining areas^22^. Xie et al. used the Fisher discriminant method to establish a regression function relationship between borehole water flow and direct current detection data, and then established a model to identify water-rich burnt rock areas. Based on the evaluation of training samples, the accuracy of the water-rich discriminant model was 89.1%^23^.

This paper comprehensively utilizes transient electromagnetic measurement and high-density electrical measurement. The object is to investigate the underground water accumulation range and burnt rock boundary of Jiangjun Gobi No. 1 open-pit coal mine. The study area is located in the Kalamaili Fault Zone, a significant large-scale fault zone in northern Xinjiang and even Central Asia. It was formed after the closure of the Paleozoic ocean basin^24^. Due to the high groundwater levels, reaching tens of thousands of cubic meters per day in some sections of the study area, it is crucial to employ advanced technical methods to accurately determine the scope and spatial distribution of groundwater. This is necessary to mitigate the significant risk factors and production disruptions it poses. The innovation of this study lies in the use of different geophysical exploration methods according to the depth of the formation, among which the transient electromagnetic method with high resolution is used in the middle and deep layers, and the high-density electrical method with high accuracy is used in the shallow parts. Among the various electromagnetic methods, the transient electromagnetic method stands out as the only electromagnetic coupling method in the time domain. It has high resolution and advanced technical methods, and can accurately determine the resistivity distribution characteristics of target layers in the middle and deep layers. The high-density electrical method compensates for the limited data obtained from shallow transient electromagnetic measurements by offering a large mount of data and employing advanced forward and inversion procedures. This paper utilizes transient electromagnetic and high-density electrical resistivity methods, the spatial shape and distribution range of the target layer can be accurately delineated. It can be used to avoid geological disaster risks and provide detailed geological basic data for project development or construction design.

## Study area

The study area of this paper is the Jiangjun Gobi No. 1 Open-pit Coal Mine field, which is located in the southeast of the Junggar Basin, Xinjiang Uygur Autonomous Region, China. The area is higher in the southeast and slightly lower in the northwest. The landform is a hilly denuded plain with an altitude of 537–616 m and a relative height difference of about 30–50 m. The ore field has a continental arid desert climate, with large annual and day-night temperature differences. June to August is summer, and the climate is hot. The daytime temperature is often above 40 ^∘^C, and the absolute maximum temperature reaches 43.2 ^∘^C on July 13, 2004. Winter is from November to February of the following year, and the climate is extremely cold, with the absolute minimum temperature reaching − 49.8 ^∘^C on January 26, 1969. The annual average precipitation is 106 mm, the annual evaporation is 1202–2382 mm, the annual sunshine is 3053 h, there are occasional thunderstorms from May to August. Snow cover is scarce in winter. The maximum snow thickness is 0.3 m and a maximum permafrost depth is 2 m. The area is windy all year round, with the wind force generally ranging from 4 to 5, and occasionally reaching more than 10, accompanied by strong sandstorms.

**Figure 1:**
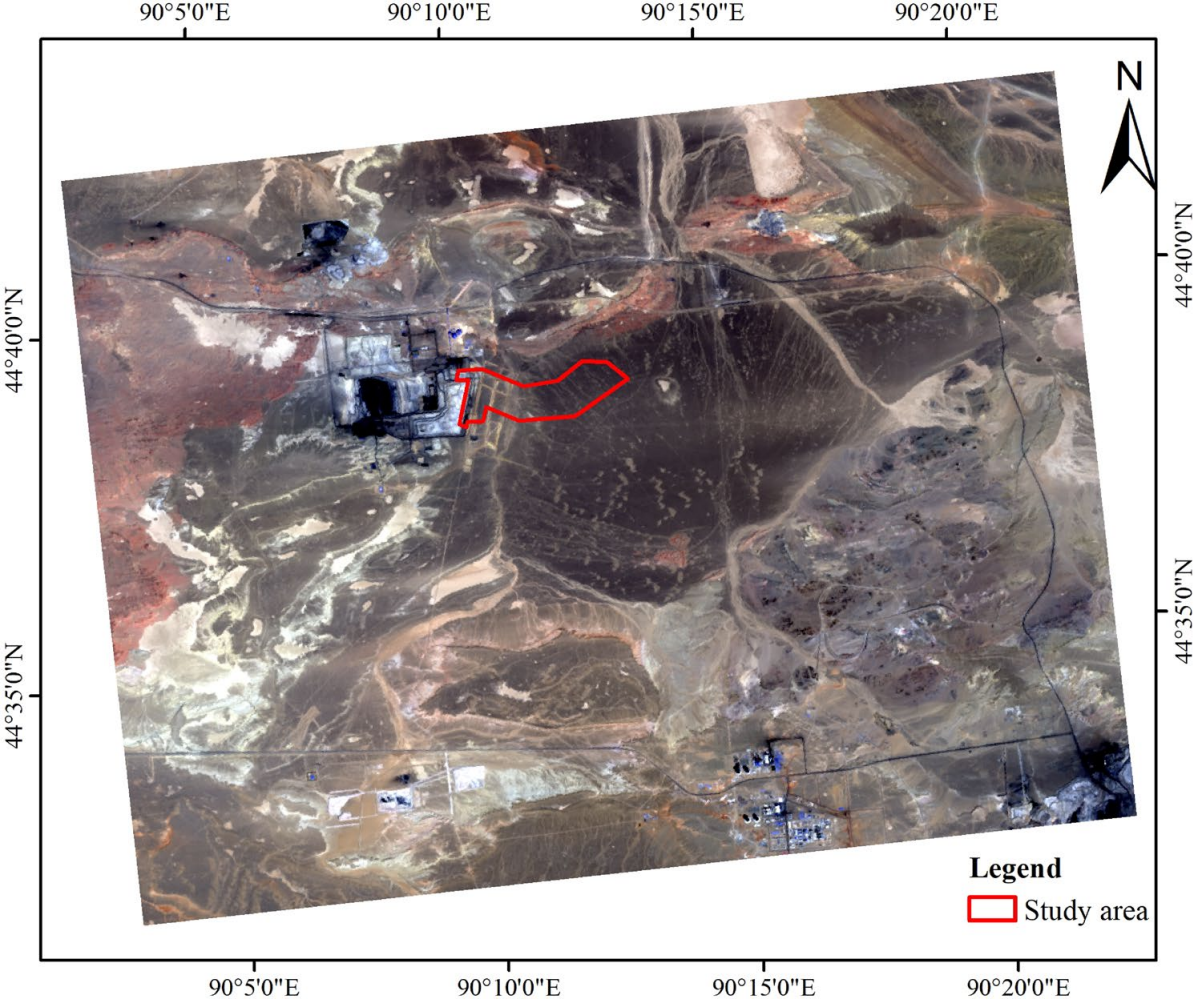
The red frame in the figure represents the extent of the study area. This figure was created using ArcGIS 10.2 geographic information software. The image depicted in the figure is a Landsat 8 remote sensing image. The red, green, and blue bands of the Landsat 8 remote sensing images correspond to bands 4, 3, and 2, respectively. By combining these bands, a true color image can be generated. The download link of Landsat 8 can be found at https://www.gscloud.cn/.

There is no perennial water flow on the surface of the area, and there are no spring water points. Most of the water flow consist of temporary water flow, which is formed by summer rainfall or occasional heavy rain. Temporary water flows either flow northwest or gather in low-lying areas, where they either infiltrate the ground or evaporate on the spot. Many silted mud slabs, commonly known as white slabs, are formed in the area. The Quaternary strata in the minefield are permeable and do not contain water, while the Jurassic strata have a low water content. The fissure water in the open-pit stope mainly comes from atmospheric precipitation and the surrounding surface water. These water sources flow to the bottom of the pit in scattered flow patterns resembling fissures. The water has slightly pressure, and the groundwater table is buried at a depth of about 15–30 m. The shallow part of the coal seam in the north of the minefield has been burned, and burnt rocks are widely distributed on the surface. The roof, floor and the surrounding rocks of coal seam have been baked by fire, altering the characteristics of the original rock and turning it into hard, broken burnt rocks with cracks and pores. The burnt rock area provides good channels for water conduction and water storage space. After excavation, the groundwater present in the burnt rock will accumulate in low-lying areas and fill the pit with water, posing a threat to future mining activities. According to the Xinjiang earthquake peak acceleration zoning map, the survey area is located in the 0.05 g earthquake peak acceleration zone, which classifies it as a VI degree seismic intensity fortified area. No major earthquakes have occurred in or around the area in the past 20 years. The scope of the study area in this paper is shown in Fig. 1.

Most areas in the north and east of the mine field are Quaternary, with some Jurassic exposed in the east and south of the mine, and Jurassic exposed at the northern edge. The Paleozoic Carboniferous strata are exposed in a small area in the southeast corner of the mine field. It is the sedimentary basement of the Jurassic strata. The lithology is composed of gray-brown, gray-purple andesitic porphyry, dacitic rhyolite, etc., with a regional thickness of 862–1477 m. The Mesozoic Jurassic system is exposed along the northern edge and in parts of the south. The rocks are mainly gray and gray-green with variegated fluvial and lacustrine deposits of grey-brown, purple-red and purple-brown. The lithology includes sandstone, mudstone, siltstone, containing silicified wood, and the bottom is a layer of conglomerate and sandy conglomerate. Thickness 40.43–646.58 m. The Cenozoic Quaternary is mainly distributed in the low-lying areas of mining fields. According to its origin, it can be divided into flood alluvium and seasonal flood retention layers. Among them, the Upper Pleistocene to Holocene alluvial sand and gravel layer is located in the northern part of the mine field. It is accumulated in the Gobi Plain and is mainly gravel, sand and a small amount of soil formed by alluvial deposits. It is loosely accumulated and has a large thickness variation, about 1–25.85 m, with an average thickness of 9.23 m. Holocene seasonal floods deposited silt, sand, sub-sand and sandy clay layers, mainly scattered in the low-lying areas of the western terrain of the mine field. It is seasonal flood retention deposits, including silt, loose silt, sand, sub-sand soil and sandy clay. It is commonly known as whiteboard due to evaporation. The thickness is generally not large, mostly within 0.5–5 m.

## Methods

In this paper, the transient electromagnetic method and high-density resistivity method are used to conduct a geological survey on the boundary of burnt rock and water accumulation area in the Jiangjun Gobi No. 1 open-pit coal mine area.

### Transient electromagnetic

The transient-electromagnetic method, also known as the time domain electromagnetic method (TEM for short), belongs to the detection method of electromagnetic induction. Its mechanism evolves the generation of an eddy current field effect produced by the conductive medium when exposed to a step-changing electromagnetic field. In other words, it utilize an ungrounded return line or a magnetic dipole (or a grounded line source electric dipole) to emit pulsed electromagnetic waves underground as the excitation field source (commonly referred to as the “primary field”). According to Faraday’s law of electromagnetic induction, after the pulsed electromagnetic wave ends, the earth or the detection target will generate an induced eddy current inside it due to the influence of the excitation field (i.e., “primary field”). This induced current exhibits both spatial and temporal characteristics. Its magnitude is influenced by various factors, such as the spatial and electrical properties of the target, the characteristics of the excitation field, and so on. Over time, the induced current gradually weakens until it dissipates due to heat loss. The secondary field signal corresponding to the primary field pulse signal can be expressed as Eq. (1):

1 $$\begin{aligned} V=\frac{\mu _0^{\frac{5}{2}}\cdot M \cdot q}{20\pi \cdot \sqrt{\pi \rho ^{\frac{2}{3}} \cdot t^{\frac{5}{2}}}} \end{aligned}$$

where $\mu _0$ represents Permeability, *M* represents the transmitting coil magnetic moment, *q* represents the receive coil equivalent area, $\rho$ represents formation resistivity, and *t* represents time.

#### Data processing workflow

- *Data synthesis*: Determine the appropriate maximum frequency point spacing and density to synthesize the original sampling data.
- *Wavelet denoising*: Apply wavelet filtering to the raw data.
- *S-transform denoising*: Apply S-transform filtering to the original data.
- *Smoothing of curves*: Inspect the attenuation curve after denoising and synthesis, and perform further smoothing as needed.
- *Terrain Effect Correction*:

From the apparent resistivity-depth profile (or the apparent resistivity plan, if necessary) and the topographic map, determine whether there is a terrain influence, if present, apply the ratio method or spatial filtering method for correct. Using specialized software, convert parameters such as $\rho _\tau (t)$ (apparent resistivity) and $H_\tau (t)$ (apparent depth). The data collected in the field by TEM exploration is induced electromotive force. The abscissa is an arithmetic coordinate indicating the time of observing the secondary field, while the ordinate is a logarithmic coordinate indicating the normalized induced electromotive force. Generally, the induced electromotive force needs to be converted into apparent resistivity, and the conversion formula is given by Eq. (2):

2 $$\begin{aligned} \rho =\frac{\mu _0}{4\pi t} \left[ \frac{2\mu _0 S_T S_R}{5t(V(t)/I)} \right] ^{\frac{2}{3}} \end{aligned}$$

where $\mu _0=4\pi \times 10^7$ H/m, $S_T$ is the area of the sending loop, $S_R$ is the area of the receiving coil, *t* is the measurement time, *V*(*t*)/*I* is the transient value of the normalized induced electromotive force. All the above quantities are expressed in accordance with the international standard units of measurement.

### High-density electrical resistivity

The high-density electrical resistivity method follows the same basic principle as the traditional resistivity method. However, it offers a denser distribution of measuring points during observations. Druing on-site measurements, electrodes are placed at regular intervals on the measuring points and observations are made. The electrodes can be flexibly combined, allowing for the extraction of more geoelectric information. Electrical prospecting can adopt a covering measurement method similar to seismic prospecting.

#### Working parameters of the device

Compared to commonly used devices such as dipoles, differentials and combined three-level devices, the Wenner quadrupole device offers advantages in terms of reduced susceptibility to surface fluctuations and greater measurement depth. Consequently, the Wenner device was utilized for this measurement. High-density electrical exploration should aim to minimize the impact of terrain undulations. However, completely avoiding them is challenging in practical work. Therefore, when conducting high-density electrical exploration, it is crucial to consider which device is least affected by terrain. Among the device types used in high-density electrical methods, the dipole device is most severely affected by terrain. Its electrical sounding curve is inherently complex, and when combined with the terrain factors, the identification of its electrical sounding profile becomes difficult. When the three-pole device encounters a valley or ridge, the electrical measurement curve exhibits multiple peaks, and the response levels of the AMN and MNB devices become uneven, making it challenging to distinguish between them. In comparison, the quadrupole device is least affected by terrain, and the shape of its electrical sounding profile is easier to interpret. Quadrupole devices can be further divided into Wenner and Schlumberger devices. The Wenner device offers relatively high vertical resolution and is sensitive to reflecting the vertical distribution of geological bodies. Therefore, the Wenner device is selected for exploration tasks that require higher vertical resolution. On the other hand, the Schlumberger device exhibits high sensitivity to changes in the horizontal direction of geological body. As a result, this device is chosen for exploration tasks that require high horizontal resolution. In conclusion, the Wenner device, which is less affected by terrain and provides higher tangential and vertical resolution, was selected for this work as described in this article.

**Figure 2:**
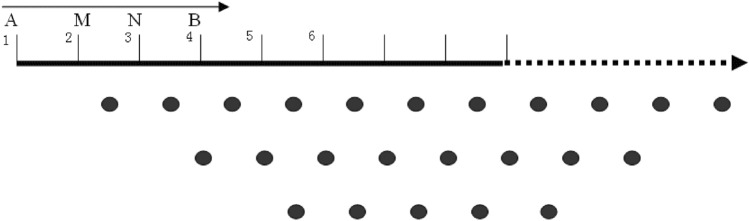
Schematic diagram illustrating the placement of measuring points in Wenner array.

In the Wenner quadrupole device, it is possible to arrange 60–120 electrodes at once, while sections exceeding 120 are measured by rolling, with a point distance of 5–10 m. This method allows for the collection of 552–2970 data points within a fixed section in a measurement (552 data points of 60 channels and 16 layers, 1275 data points of 90 channels and 25 layers, 2025 data points of 120 channels and 25 layers, and 2970 data points of 180 channels and 20 layers). The schematic diagram of Wenner quadrupole device is shown in Fig. 2.

#### Technical process

1. Data collection
  At the measurement point, drive the copper electrode into the ground to a depth of 15 cm.
2. Data processing and inversion
  Data preprocessing To enhance the inversion accuracy and interpretation effectiveness, the measured apparent resistivity data need to undergo preprocessing. The raw measurement data is processed using the RES2DINV software from Sweden. The processing steps include: Filtering—automatically remove data outliers using the median filtering method or manually removing outliers through graphical interaction.
  Terrain correction Load and edit terrain data from a text file. Set the maximum number of correction interactions to three, then initiate terrain correction to account for the deviations caused by terrain effects.
  Inversion Perform 2D inversion using the Swedish RES2DINV software. The inversion utilizes the apparent resistivity cross-section georeformation data as the initial model and completes after several iterations, indicated by a well-converged convergence curve.

## Experiments

### Transient electromagnetic method

#### Survey line distribution map

**Figure 3:**
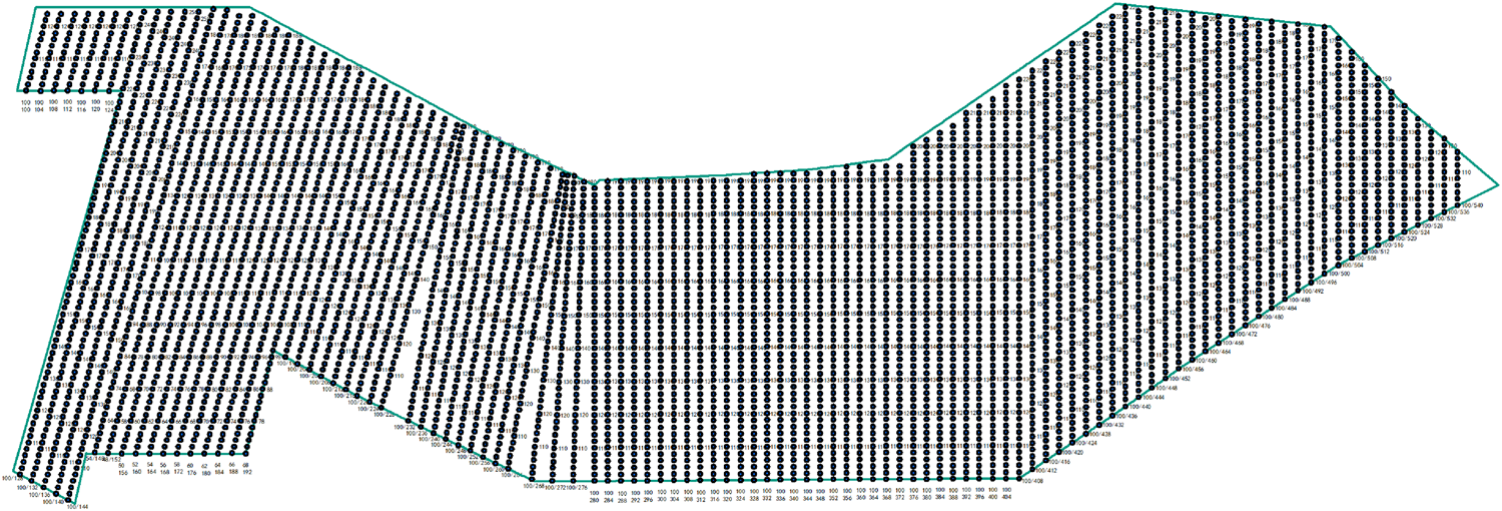
The spatial distribution of the measuring lines.

The spatial distribution of the measuring lines is shown in Fig. 3.

#### Quality inspection

The accuracy classification of quality inspection is shown in Table 1.

**Table 1:** Accuracy classification table.

Class	Mean square relative error/%	
	The point error	The point-free error
I	10	5
II	15	10

The point-free error is the result of combining the observation error of *B* or *dB*/*dt* and other errors, including those caused by variations in external electromagnetic noise and instrument instability.

The point error is the combination of the device error and point-free error.

The device error arises from geodetic inaccuracies, as well as imprecise wiring and point placement.

The quality of data in the entire region is measured by the overall mean square relative error M, and its calculation formula is as follows:

3 $$\begin{aligned} M=\pm \sqrt{{\frac{1}{2nm}}\sum _{(i,j=1)}^{nm}\left[ \frac{V_{j}(t_i)-{V^{\prime }_j}(t_i )}{\overline{V_j} (t_i )} \right] ^2} \end{aligned}$$

In the formula, *n* represents the number of test channels participating in statistical calculation. *m* represents the number of detection points. $V_j(t_i)$ represents the original observation data of j-th point and i-th measurement track. $V_j^{\prime }(t_i)$ represents the j-th point i-th track system inspection observation data. $\overline{V_j} (t_i )$ represents the average value of $V_j(t_i)$ and $V_j^{\prime }(t_i)$.

We have completed a total of 108 transient electromagnetic measurement lines. We have a total of 5285 measurement points, with 5112 points related to production and 173 points inspected for quality, all of which are qualified. The quality inspection rate is 3.17%, and the mean square relative error of the inspected points across the entire measurement area is 6.38%. The measurements meet Class I standards, which align with the design and specification requirements. Table 2 displays the statistical data of transient electromagnetic checkpoints in the entire area.

**Table 2:** Transient electromagnetic quality inspection statistics table.

Work method	Physical points	Detection points	Quality inspection rate	Accuracy	Remark
Transient electromagnetic	5112	173	3.17%	6.38% (Class I)	Total mean square relative error

#### Interpretation of typical sections

**Figure 4:**
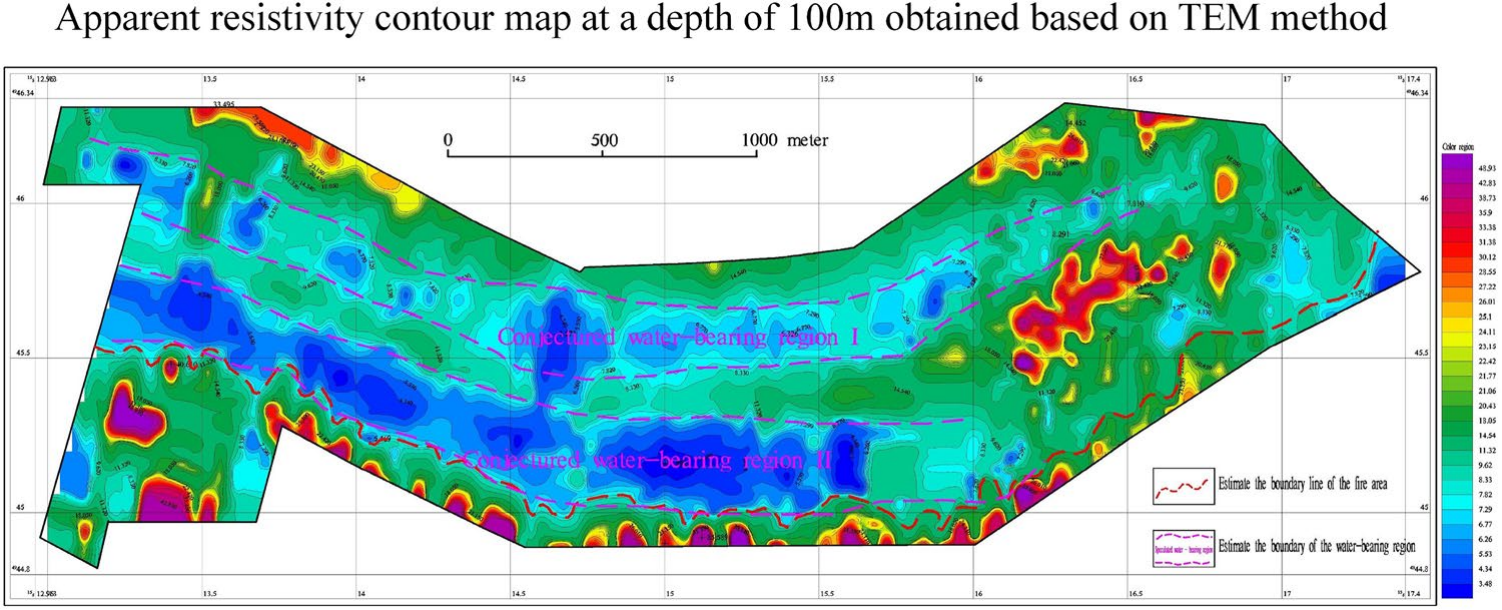
A contour map of Apparent resistivity at a depth of 100m obtained using the TEM method. This figure was generated in GEOIPAS 4.6 software. The official website address of GEOIPAS software is https://www.jinweisoft.com/f/list-b4bb0d91026d47128d9755d15253eba7.html.

To provide a more intuitive depiction of the vertical electrical changes and accurately identify the boundary of the burnt area and the location of local water distribution on the plane, data slices of different depths are extracted from the transient electromagnetic data in the vertical direction. This study analyzes the apparent resistivity plan diagrams at depths of 45 m, 100 m, 150 m, and 240 m. By comparing and analyzing the electrical characteristics of planes at different depths, it is possible to infer the boundary of the burnt area and the location of water accumulation. This analysis primarily focuses on the interpretation and inference of the apparent resistivity plan at a depth of 100 m. Figure 4 illustates the plan view of the apparent resistivity contours of the TEM at this specific depth. The low resistivity range is between 10 and tens of $\Omega \cdot$ m, the medium resistivity range is between 100 and 400 $\Omega \cdot$ m, and the high resistivity is defined as values greater than 500 $\Omega \cdot$ m. Based on the transient electromagnetic results, the electrical characteristics exhibit a pattern of medium and high resistance-low resistance-medium resistance-low resistance-high resistance from north to south on the map. The eastern and western regions of the map show relatively medium and high resistivity, suggesting a normal formation or a fire zone without water. Figure 4 clearly indicates the presence of significant low-resistance anormaly zones on the northern and southern sides of the study area. After the coal seam is burned, the electrical characteristics of the normal formation are disrupted by the local areas with high water content, and the apparent resistivity is characterized by low resistance. It is speculated that there are two water-rich areas (blue areas in the Fig. 4), labeled as I and II from north to south. The low-resistivity anomalies, indicating better local traps of water, are presumed to correspond to regions with higher water content. Based on geophysical characteristics, the electrical characteristics of the coal seam exhibit medium to high resistance, leading to the conclusion that the boundary of the burning area is located on the south side of the study area (indicated by the red dotted line).

**Figure 5:**
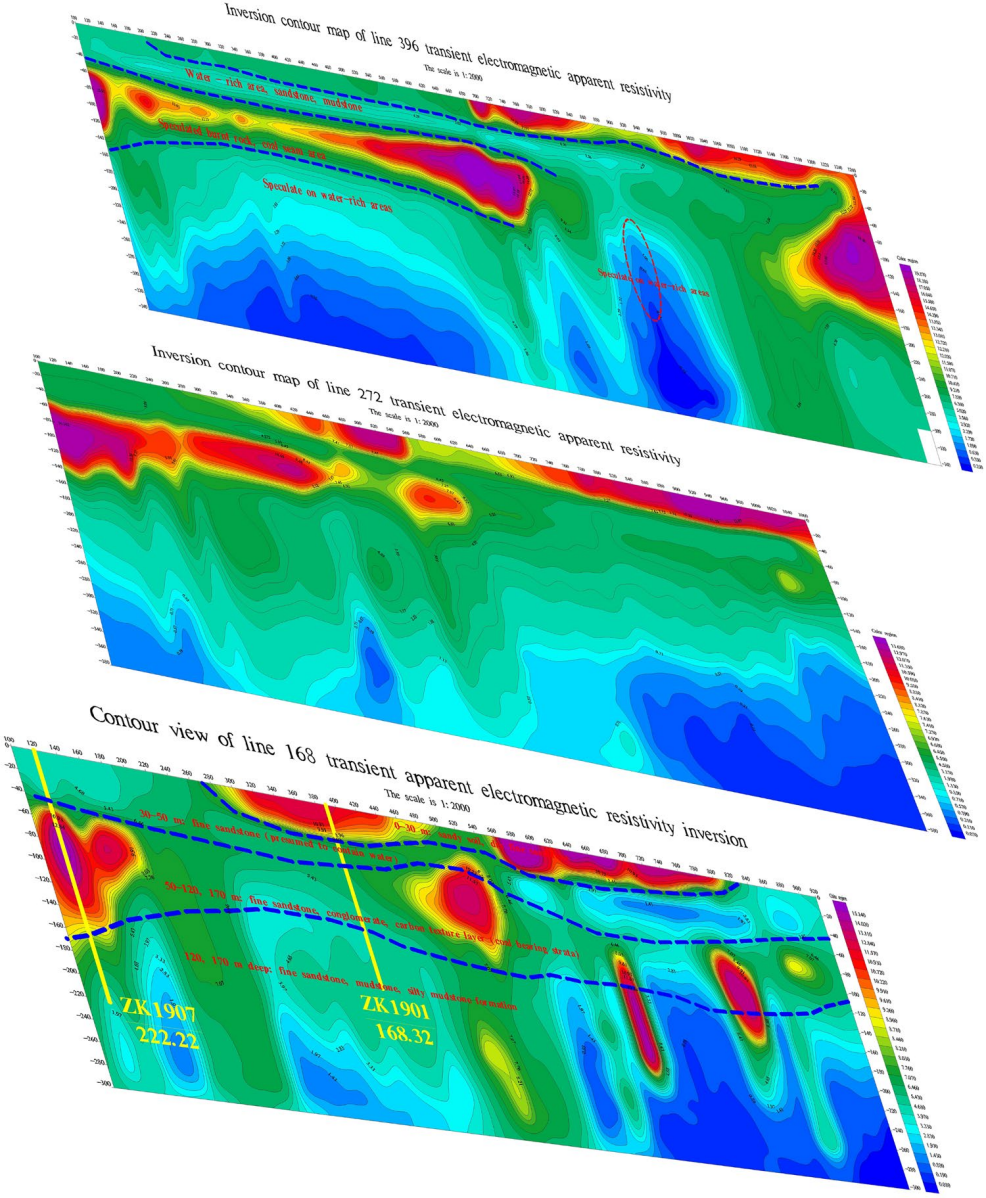
Cross-sectional characteristic diagrams of different survey lines using the TEM method. This figure was generated in GEOIPAS 4.6 software. The official website address of GEOIPAS software is https://www.jinweisoft.com/f/list-b4bb0d91026d47128d9755d15253eba7.html.

This study selected representative sections for a comprehensive analysis of the vertical electrical characteristics. Figure 5 illustrates the vertical section characteristics of the combined TEM sounding profile. The overall vertical electrical characteristics of the survey area exhibit similarities. The electrical characteristics of the section are divided into four layers, with the electrical properties ranging from high resistance-medium, low resistance-high resistance-medium and low resistance in a shallow-to-deep sequence. The shallow part is characterized by relatively high resistivity, which is attributed to the presence of dry sandy soil and fine sandstone. The region between 20 and 45 m from surface exhibits low-resistivity, displaying significant changes in contour density. It is speculated that this low-resistivity anomaly is caused by water-bearing sandstone and argillaceous siltstone. At a depth of approximately 40–140 m, there is a relatively high-resistivity region, which is presumed to be attributed to burnt rock areas or coal-bearing strata. The high-resistivity region teminates with an irregular low-resistivity anomaly, and the irregular contour lines are believed to be a result of the inhomogeneity of the coal-bearing strata. The deep section of the profile reveals a low-resistivity anomaly, which is likely caused by siltstone, fine sandstone formations, and water-bearing burnt rock areas. The siltstone and fine sandstone layers exhibit low resistivity with relatively gradual contour changes. Due to the presence of groundwater in the burned area, the resistivity is lower than that of the surrounding formations, resulting in distorted and densely changing contour shapes. This phenomenon is indicative of a water-enriched burned area and serves as an important criterion for distinguishing between normal strata and water-bearing burnt rock areas.

**Figure 6:**
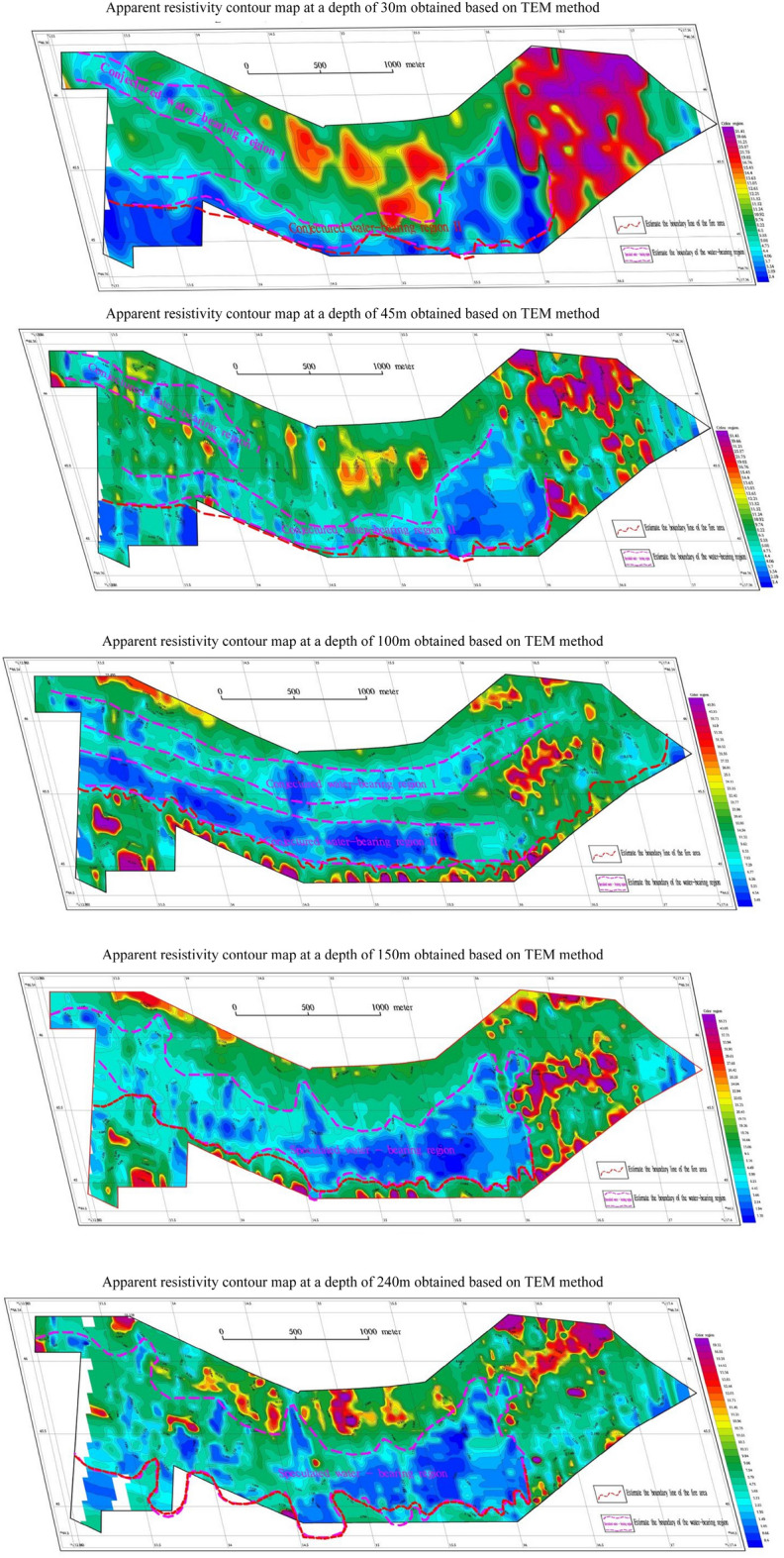
Contour maps of apparent resistivity at various depths were generated using the TEM method. This figure was generated in GEOIPAS 4.6 software. The official website address of GEOIPAS software is https://www.jinweisoft.com/f/list-b4bb0d91026d47128d9755d15253eba7.html.

Figure 6 shows transient electromagnetic slices at different depths. Based on Fig. 6, it can be concluded that the apparent resistivity of slices at depths of 30 m, 45 m, 100 m, 150 m, and 240 m is similar from shallow to deep, and the low-resistance anomaly area gradually increases from shallow to deep. The 45-m iso-depth apparent resistivity plan reveals that the local low-resistivity areas on the north and south sides of the study area are water-filled, and the contours are trapped and highly variable. The eastern part of the study area exhibits relatively high resistivity, suggesting an unwatered formation in the burned area. As the depth increases, the low-resistivity area gradually expands. The 100-m iso-depth slice map shows two distinct low-resistivity anomalies, which can be inferred as water-rich areas. In the 150-m iso-depth slice map, the low-resistance anomalies on the north and south sides gradually converge towards the middle-south. By analysing the morphological characteristics of the contour lines, regions where the contour lines change more intensively are identified as locally water-bearing areas, while areas with gentler contour line changes are speculated to be caused by the low-resistance anomalies resulting from argillaceous siltstone strata. The eastern region exhibits a relatively high-resistivity anomaly, which is presumed to be an anhydrous formation or a burnt area. The low-resistance anomaly area in the 240-m iso-depth slice map is similar to the 150-m low-resistance anomaly area. The areas where the low-resistance contour lines change intensively are presumed to be caused by the water-containing burning area, while the low-resistance anomaly area with more gradual contour line changes are attributed to mud, siltstone, sandstone and other strata. The boundary of the burned area is deduced from the division of dense contours of low-resistance anomalies and high-resistance anomalies. The boundary of the 45-m deep burning area is presumed to be located on the south side of the survey area, close to the southern end of the survey line. For the 100-m depth, the boundary of the burnt area is estimated to be about 90–167 m on the north side near the southern endpoint, based on the anomalous characteristics of high and low resistance and the dense contours. The boundary of the burned area at a depth of 150 m is similar to that at a depth of 100 m. For the 240-m depth, the boundary of the burning area is estimated to be located on the south side of the study area, based on the anomalous characteristics of high and low resistance and the dense contour line.

### High-density electrical method

#### Survey line distribution map

**Figure 7:**
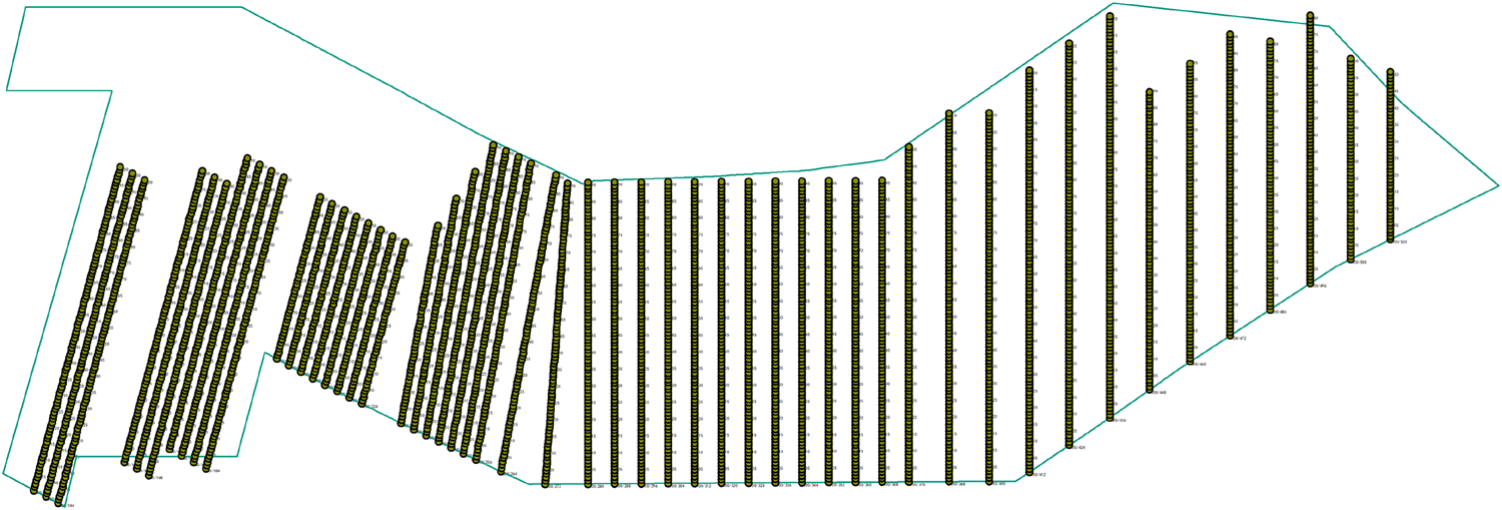
The spatial distribution of the measuring lines.

The spatial distribution of the measuring lines is shown in Fig. 7.

#### Quality inspection

52 sections were actually measured, with a total section length of 45,100 m and a total of 4510 physical points. The quality inspection section had a length of 1700 m and 170 physical points, accounting for 3.77% of the total, meeting the requirement of 3–5% specified. The relative mean square error of the profile is 0.05%, which satisfies the specification requirements by being less than ± 1.5%, achiving Class I precision. Table 3 shows the statistical data for the High-density measurement checkpoints in the entire area.

**Table 3:** High-density measurement quality inspection statistics.

Work method	Physical points	Detection points	Quality inspection rate	Quality inspection accuracy	Remark
High density electrical method	4510	170	3.77%	0.05% (Class)	No bit mean square relative error

**Figure 8:**
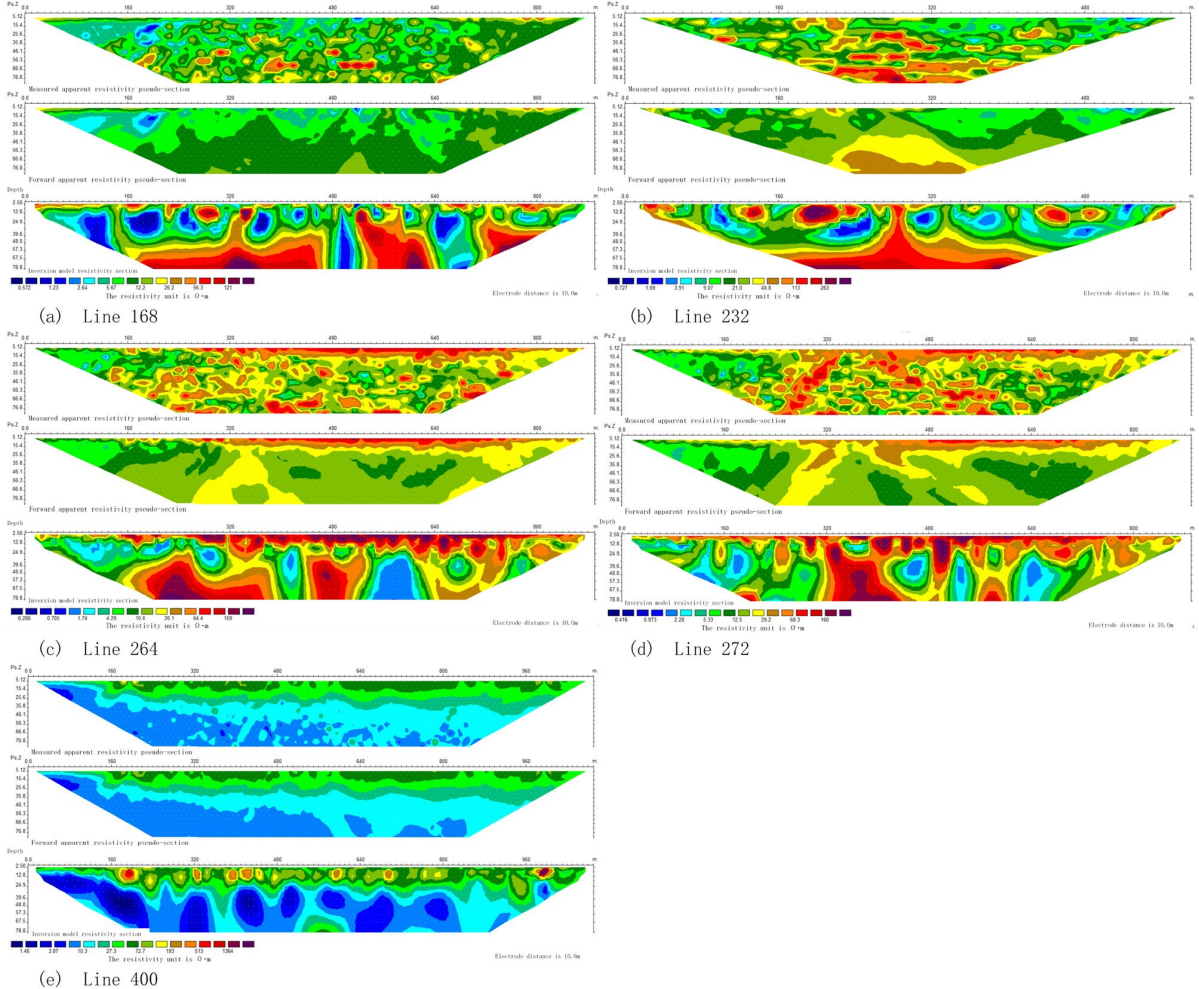
Resistivity forward modeling and inversion of the five measuring lines. This figure was generated in GEOIPAS 4.6 software. The official website address of GEOIPAS software is https://www.jinweisoft.com/f/list-b4bb0d91026d47128d9755d15253eba7.html.

The results of resistivity forward modeling and inversion for the five measuring lines are represented in Fig. 8.

1. Line 168

The detection depth of the entire section is about 80 m, and the apparent resistivity ($\rho s$) varies between 1.08 and 400.21 $\Omega \cdot$ m. The surface of the section is covered by Quaternary sedimentary layer. Due to the presence of the coal mining activities, some areas are densely covered with coal ash layer, ranging from 10 to 20 cm in thickness. The entire section is situated on the western side of the study area. Judging from the overall shape of the section, the geological boundaries are relatively well-defined. Locally, there are some spherical and elliptical bodies with high-resistivity, which may be attributed to gravel layers or shallow irregular dry mudstones. A low-resistivity layered anomaly is observed within a depth of 50 m at the 10-m position of the survy line, which is likely to be a loose sandy conglomerate aquifer. Below 50 m, a high-resistance layered anomaly is present. Based on its extent, it is inferred that this anomaly may indicate a burnt rock (coal-bearing stratum) area. At 460–520 m and 620–720 m of the survey line, the low-resistance anomaly continues to extend deeper, possibly due to the influence of small fracture zones between the layers. Water from the aquifer infiltrates along the fracture surfaces, spreading to greater depth and causing the low-resistance anomalies to extend in a band shape.

2. Line 232

It can be observed from Fig. 8 that the detection depth of the entire section is approximately 80 m, and the apparent resistivity ($\rho s$) varies between 1.08 and 335 $\Omega \cdot$ m. Due to the coal mining, the coal ash layer is thicker, ranging from 10 to 20 cm. This section is situated in the middle-western part of the study area. Judging by the overall shape of the section, the geological boundaries are relatively distinct. Locally, there are some spherical and elliptical bodies with medium-high resistance. They are believed to be caused by gravel layers or shallow irregular dry mudstones. Low resistance strips are observed in certain areas, which are speculated to be result from structural fissure water. An anomaly low-resistivity layer appears within a depth of 50 m at approximately 20 m of the survey line, which is presumed to be loose sandy conglomerate aquifer. Below 60 m, there is a high-resistance layered anomaly. Based on its extent, it can be inferred that this anomaly may be a burnt rock (coal-bearing stratum) area. At 100 m and 200–260 m along the survey line, the low-resistance anomaly continues to extend deeper. This extension is presumably caused by the influence of small fracture zones between layers. The water in the aquifer penetrates along the fracture surface and spreads to greater depth, resulting in the extension of low-resistance anomalies in a band shape.

3. Line 264

As shown in Fig. 8, the detection depth of the entire section is about 80 m, and the apparent resistivity ($\rho s$) varies between 2.08 and 282 $\Omega \cdot$ m. The surface of the section is covered by Quaternary sediments. Due to the influence of the mined coal field, the coal ash layer is thickly covered in some places, about 10–15 cm. This section is located in the middle-to-west of the study area as a whole. Judging from the overall shape of the section, the geological boundaries are relatively clear. Some spherical and elliptical medium-high resistance bodies appear locally. These bodies are speculated to be caused by gravel layers or shallow, irregular, dry mudstones. A low-resistivity layered anomaly appears within a depth of 50 m at about 20 m of the survey line, which is speculated to be a loose sandy conglomerate aquifer. Below 50 m, a high-resistance layered anomaly is observed. Based on its extension state, it is inferred that this anomaly may be a burnt rock (coal-bearing stratum) area. At positions 380–420 m, 540–600 m, and 750–790 m along the survey line, the low resistance anomaly continues to extend deeper. This is speculated to be due to the influence of small fracture zone between layers. Water from the aquifer infiltrates along the fracture surface and spread to greater depth, resulting in the low-resistance anomaly extending in a band shape at greater depth.

4. Line 272

As shown in Fig. 8, the detection depth of the entire section is approximately 80 m. The apparent resistivity ($\rho s$) ranges between 1.46 and 234.03 $\Omega \cdot$ m. The section is situated in the central and western part of the study area, which is covered by the shallow Quaternary system. It is distributed along the low-lying areas of the Gobi, where Haloxylon ammodendron vegetation is abundant locally. The overall section shape reveals well-defined geological boundaries in this area are, suggesting a relatively stable geological structure. From 0 to 320 m along the survey line, at a depth of 10–15 m, there is a dry Quaternary overburden exhibiting medium to high resistance and a layered abnormal shape. Additionally, from 320 to 900 m along the survey line, the depth of 0–30 m showns medium to high resistance and a layered abnormal shape, which is attriuted to the presence of Quaternary deposits, surface sandstone and sand soil. Between 320 and 400 m along the survey line, at a depth of 20 m and deeper, a layered high ancestor layer is observed, likely caused by the irregular distribution of dry mudstone, burnt rock, and sandy conglomerate layers (coal-bearing strata) within the sandstone.

5. Line 400

As can be seen from Fig. 8, the detection depth of the entire section is about 80 m. The apparent resistivity ($\rho s$) varies between 1.08 and 400.21 $\Omega \cdot$ m, and the ground surfaces is covered by a Quaternary sand and gravel covering layer. Based on the overall shape of the section, the geological boundaries are clearer, and the geological structure appears relatively stable. In the section at a depth of 0–30 m, there is a dry Quaternary overburden characterized by medium to high resistivity, exhibiting distinctive layering. Moving eastward (at the end of the section), the thickness of the medium to high resistive layer increases due to the thickening of the Quaternary overburden. At a depth of 30 m, a relatively low-resistivity layer with distinct layering is observed, likely attributed to the presence of water-bearing sandstone and fine sandstone.

#### Determining the burnt rock boundary and underground water extent

**Figure 9:**
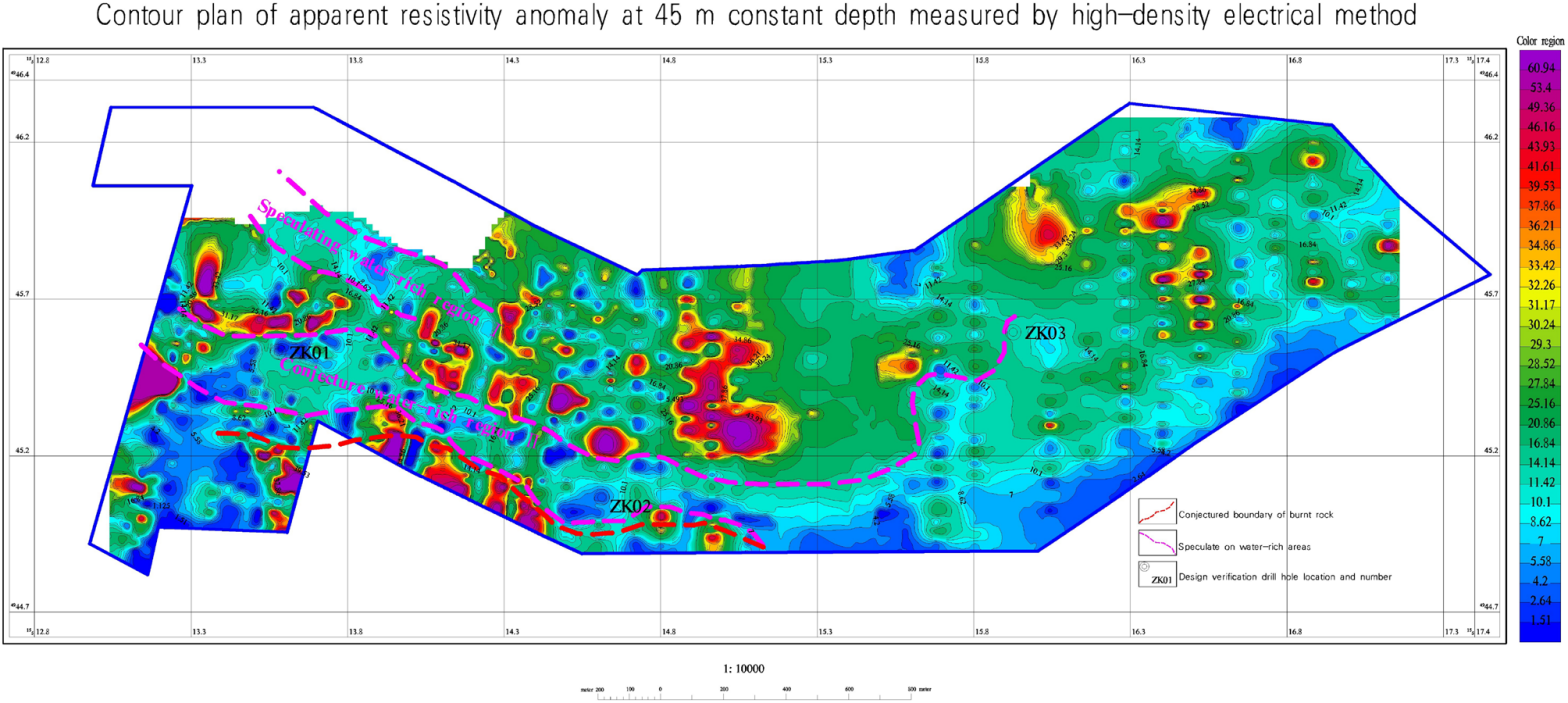
Inferred burnt rock boundary and underground water accumulation area. This figure was generated in GEOIPAS 4.6 software. The official website address of GEOIPAS software is https://www.jinweisoft.com/f/list-b4bb0d91026d47128d9755d15253eba7.html.

Base on the observed trend of apparent resistivity at a depth of 45 m and the criterion of apparent resistivity $\le 16\Omega \cdot$ m, within the area, two anomalous areas with low resistivity (labeled as I and II) were identified at the depth of 45 m, as shown in Fig. 9.

No. I low-resistivity zone is located in the northwest of the study area and continues to extend to the northwest without traps. The deep low-resistivity zone mainly consists of sandstone and coarse sandstone. Due to the influence of surface water, it exhibits low resistance, suggesting it is a area with abundant water. No. II low-resistivity area is situated in the southern part of the study area, extending to the east and west sides without traps. The deep low-resistivity area is mainly composed of sandstone, coarse sandstone, and burnt rock, with intermittent layers of mudstone and argillaceous sandstone. Influenced by the supply water, it presents a low resistance, which is presumed to be a water-rich area. The boundary of burnt rock (coal-bearing strata) is located in the south of the study area. The resistivity of burnt rock (coal-bearing strata) is higher than that of surrounding rocks, and the resistivity of coal seams is also slightly higher than that of sandstone with higher porosity. It is inferred that the high-resistance boundary in the south marks the southern limit of burnt rocks, while the water-rich boundary in the north indicates the northern boundary of burnt rocks. The surrounding rocks have gone baking due to the spontaneous combustion of coal seams, and water or muddy substances have infiltrated through the porosity, resulting in an overall low resistance. The high-resistivity formations in the south and north of the study area may be interbedded with coal-bearing strata and coarse sandstone.

## Conclusions

Transient electromagnetic and high-density resistivity methods can be applied to geological surveys, prediction of burnt rock boundaries, and prediction of groundwater in subsurface exploration. By conducting investigations on the range of underground water and burnt rock boundaries in the Jiangjun Gobi No. 1 Open-pit Coal Mine, we summarized the electrical characteristics of the study area and approximately determined the boundaries of underground water and burnt rock. This provides reference data for avoiding geological hazards, mineral development, and ecological environment protection.

The Jiangjun Gobi No. 1 open-pit coal mine is located in the Kalamaili fault zone and is rich in groundwater. Different geophysical exploration methods are employed in this paper according to the depth of the formation. Among them, the transient electromagnetic method with high resolution is used in the middle and deep parts, while the high-density electrical method with high accuracy is used in the shallow parts. By studying the geophysical data, this paper addresses several fundamental geological issues related to the distribution of burnt rock boundaries and groundwater in the Jiangjun Gobi No. 1 Open-pit Coal Mine. Based on the measurement results, two water-rich areas (labeled as I and II in Figs. 6 and 9) were inferred, and the boundaries of burnt rock at different depths were identified.

The study of the geophysical data and geological information of the entire study area leads to a systematic understanding of the stratigraphy and electrical distribution characteristics of the study area, as follows:

1. The geological structure throughout the study area is weakly developed yet relatively stable. The overall strata exhibit low-resistance characteristics, with relatively low resistivity values. In this transient electromagnetic work, the boundary of the burned area and the water-rich burnt rock area were inferred based on abnormal relative high or low resistance and the shape and characteristics of the resistivity contour.
2. The relatively high-resistance layer near the surface is caused by the fourth-grade sand and gravel layer.
3. A low-resistance layer exists within a range of 20–50 m throughout the study area, which is inferred to be an aquifer. Due to the local presence of interlayer fracture zones, surface water seeps downwards, and the low-resistance layers tend to connect to deeper layers. It is speculated that the relatively abnormally high resistivity in the middle and deep depths is caused by burnt rocks and coal-bearing formations. The anomalous low resistivity in the deep is caused by sandstone and water-rich burnt rock.
4. Based on the results of the transient electromagnetic and high-density resistivity methods, two groundwater areas eastward (labeled as I and II in Figs. 6 and 9) were inferred.
5. The boundaries of the burnt area at depths of 45 m, 100 m, 150 m, and 240 m were inferred. The burnt rock boundary is inferred to be the boundary between the relatively high-resistance and low-resistance areas in the southern and entire northern parts of the study area, which is the boundary between the inferred water-rich area and the relatively high-resistance area.

In this geophysical exploration, validation boreholes were established in the corresponding areas based on the distribution of groundwater and burnt rocks. The validation boreholes were all vertical boreholes. It is suggested to verify the inferred burnt rock and water-rich boundaries within the study area based on the design borehole depth and location, to provide technical support for other engineering projects through constrained inversion of the entire geophysical exploration results. According to prior experience, the high-resistance area may be formed by interlayers of burnt rocks, coarse sandstones, and other layers generated by coal spontaneous combustion and baking, and there may also be coal-bearing strata. Therefore, it is recommended to strengthen validation in the future. Although this geophysical exploration achieved certain exploration objectives, it cannot replace the engineering geological survey or related evaluation work in the later development and construction stages. It is recommended to conduct engineering surveys of rocks, soils, hydrology, and other aspects in the entire study area before engineering construction and to conduct engineering geological surveys or related evaluations in each stage according to relevant specifications.

## Data Availability

The datasets generated and analysed during the current study are not publicly available since the data belongs to the mine resource management unit, the authors do not have the authority to disclose the data. However, the data is available from the corresponding author upon reasonable request.
